# Comparative efficacy of modified FOLFIRINOX, gemcitabine plus capecitabine and gemcitabine plus nab-paclitaxel as adjuvant treatment for resected pancreatic cancer: a Bayesian network meta-analysis

**DOI:** 10.3332/ecancer.2021.1276

**Published:** 2021-08-16

**Authors:** Victor Hugo Fonseca de Jesus, Rachel P Riechelmann

**Affiliations:** Medical Oncology Department, A.C. Camargo Cancer Center, Rua Prof. Antônio Prudente 211, São Paulo SP 01509-010, Brazil

**Keywords:** FOLFIRINOX, gemcitabine, capecitabine, nab-paclitaxel, adjuvant, pancreatic, cancer

## Abstract

**Background:**

There are no head-to-head comparisons evaluating the efficacy of the main polychemotherapy regimens used for patients with pancreatic cancer in the adjuvant setting. We aimed to describe the relative efficacy of modified FOLFIRINOX (mFOLFIRINOX), gemcitabine plus capecitabine (GEM-CAP) and gemcitabine plus nab-paclitaxel (GEM-NAB) in this setting using a Bayesian network approach.

**Methods:**

We collected data from the ESPAC-4, PRODIGE 24 and APACT trials. Disease-free survival (DFS), according to the investigators, and overall survival (OS) for the three polychemotherapy regimens were compared using gemcitabine as the reference arm. We ran Markov chain Monte Carlo simulations with a fixed-effect model to generate the posterior distribution of the hazard ratios (HRs) using non-informative priors. Relative efficacy was measured by HRs, surface under cumulative ranking and rankograms.

**Results:**

mFOLFIRINOX was the chemotherapy regimen most likely to be the most effective in the adjuvant setting (98.9% and 89.6% probability for DFS and OS, respectively). GEM-NAB marginally improved DFS (HR = 0.97, 95% credible interval (95% CrI) = 0.77–1.21) and OS (HR = 0.98, 95% CrI = 0.76–1.25) when compared to GEM-CAP. However, GEM-NAB had the highest chances of being the second most active chemotherapy regimen (61.4% and 52.5% probability for DFS and OS, respectively), whereas GEM-CAP was less likely to represent the second most active regimen (37.7% and 40.1% probability for DFS and OS, respectively).

**Conclusion:**

For patients eligible and fit enough to undergo adjuvant treatment with mFOLFIRINOX, this constitutes the treatment of choice. For those with contraindications to mFOLFIRINOX, while both GEM-NAB and GEM-CAP can be considered appropriate alternatives, GEM-NAB is likely the most effective regimen.

## Background

Pancreatic cancer currently stands as the 14th most common type of cancer worldwide, with 458,918 estimated cases in 2018 [1]. In the same year, it represented the seventh most common cause of cancer-related mortality, with 432,242 estimated deaths. Furthermore, data from developed [2, 3] and developing [4] countries suggest that the burden of pancreatic neoplasms in cancer epidemiology is expected to grow significantly in the decade to come. This high lethality rate is partially related to the disease stage at diagnosis. Only 15%–20% of the patients are candidates for curative-intent surgery [5]. Moreover, for those submitted to surgery, survival outcomes are dismal when this treatment modality is used in isolation [6].

In this sense, the ESPAC-1 [7] and the CONKO-001 [8, 9] have established the role of 5-Fluorouracil and gemcitabine in the treatment of localised pancreatic cancer, respectively. Subsequently, these two drugs were compared in the ESPAC-3 trial [10]. The authors demonstrated that while there were no differences in survival, gemcitabine was associated with lower rates of treatment-related serious adverse events (7.5% versus 14.0%). As a result, gemcitabine became the standard chemotherapy regimen in this scenario.

With the rise of polychemotherapy regimens such as FOLFIRINOX and gemcitabine-based combinations in the metastatic setting [11–13], randomised trials were designed to assess whether patients could benefit from treatment intensification in the setting of less advanced disease. While two randomised trials showed no benefit of adding erlotinib to gemcitabine [14, 15], three randomised trials demonstrated improved overall survival (OS) with combination chemotherapy: the ESPAC-4 (gemcitabine plus capecitabine (GEM-CAP)) [16], the PRODIGE 24 (modified FOLFIRINOX) [17] and the APACT (gemcitabine plus nab-paclitaxel (GEM-NAB)) trials [18].

At this moment, there are no randomised trials directly comparing these three chemotherapy regimens and such comparisons are unlikely to be ever pursued in a controlled setting. Thus, we analysed the results of these trials using a Bayesian network meta-analysis framework. This methodology allows us to infer the probable gains (e.g., in OS) of an intervention over another when formal direct comparisons of therapies through randomised trials are not available. An advantage of this approach is that one can generate probabilistic results that can support the decision-making process [19]. Our aim was to establish the relative efficacy and to compare the toxicity profile of each of these chemotherapy regimens using single-agent gemcitabine as the reference treatment.

## Methods

### Data extraction and collection

Data regarding the inclusion and exclusion criteria, the characteristics of the studies’ populations and the toxicity profile were extracted from the original publications for the ESPAC-4 [16] and PRODIGE 24 [17] trials and from the 2019 American Society of Medical Oncology Annual Meeting presentation for the APACT trial [18]. Additionally, updated survival data were extracted from recent presentations for the ESPAC-4 [20] and the APACT trials [21].

### Treatment schedules

In the experimental arm of the ESPAC-4 trial, patients received capecitabine 1,660 mg/m^2^ for 21 days followed by a 7 days’ rest plus gemcitabine 1,000 mg/m^2^ once a week for three every 4 weeks. In the PRODIGE 24 study, patients assigned to combination chemotherapy received 5-Fluorouracil 2,400 mg/m^2^ (46-hour continuous infusion), irinotecan 150 mg/m^2^, oxaliplatin 85 mg/m^2^ and folinic acid 400 mg/m^2^ once every 2 weeks. In the experimental arm of the APACT trial, patients received nab-paclitaxel 125 mg/m^2^ and gemcitabine 1,000 mg/m^2^ once a week for three every 4 weeks. In the control arms of the trials, patients received gemcitabine 1,000 mg/m^2^ weekly for three every 4 weeks. For all treatments, the planned adjuvant treatment duration was 24 weeks.

### Outcomes

The primary outcome was OS in the intention-to-treat population. In all three trials, it was defined as the time from randomisation to death. It was the primary outcome of the ESPAC-4 and the PRODIGE 24 trials. As a secondary outcome, we analysed investigator-assessed disease free-survival (DFS) (relapse-free survival in the ESPAC-4) in the intention-to-treat population. In all three trials, it was defined as the time from randomisation to death or disease progression. It was a secondary outcome of the ESPAC-4 and PRODIGE 24 trials, and it was a pre-specified sensitivity analysis of the primary outcome of the APACT study (independently assessed DFS). Since comparable depiction of either any severe or any grade 3–4 adverse events across the three studies was not available, we did not perform a Bayesian network meta-analysis on toxicity. Therefore, data on toxicity are summarised using only descriptive statistics.

### Statistical analysis

Before data analysis, we assessed the transitivity assumption by inspecting the characteristics of the populations and the inclusion and exclusion criteria of the studies [22]. Data on time-to-event outcomes (DFS and OS) were extracted using hazard ratios (HRs) and their 95% confidence intervals (CIs) (95% CI). Before data manipulation, we used the approach described by Parmar *et al* [23] to convert these measures into log(HR) and selog(HR), respectively. In the Supplementary Material, we have provided formulas and model specifications in accordance with Hu *et al* [24]. Given the limited number of studies and the indirect nature of all the comparisons between polychemotherapy regimens, there would be no information on between-trial heterogeneity for pairwise comparisons. Thus, we employed a pragmatic approach in which we fit a fixed-effect model to the data [25]. We used a Markov chain Monte Carlo (MCMC) method using Gibbs’ sampling algorithm to perform network meta-analyses (codes are available in the Supplementary Material). Briefly, using non-informative priors (mean equal to 0 and variance equal to 1,000) and after a burn-in phase of 40,000 iterations using three chains, we drew 120,000 samples from the posterior distribution (40,000 additional iterations for each chain without thinning). We chose non-informative priors so that results of the analyses would be driven solely by the data from the studies. Parameter convergence was checked by inspection of the Brooks–Gelman–Rubin plots [26]. Gemcitabine was used as the reference comparator (common to all trials). Pairwise comparisons were summarised using HRs and 95% credible intervals (95% CrIs). By definition, the true value of the parameter (HR in this case) has a 95% probability of belonging to such an interval [27]. To portray effectiveness, we ranked treatments according to surface under cumulative ranking (SUCRA) and plotted treatment ranks against their probabilities using rankograms [28]. These probabilities were calculated as the ratio between the number of cycles of the MCMC in which a specific treatment had a log HR (effect size) more or less than the ones of other treatments and the total number of cycles of the MCMC. We compared the distribution of categorical variables across studies with Fisher’s exact test or chi-square test. Bayesian analyses were conducted using the software WinBUGS version 1.4.3 (MRC Biostatistics Unit, Cambridge, UK). The comparisons of categorical variables across studies and the graphics were carried out using the software R version 3.6.0.

## Results

**Figure 1.**
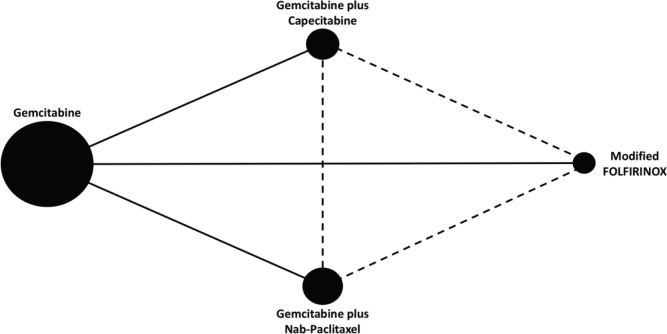
Network map. Each node represents a chemotherapy regimen. The node size reflects the total number of patients treated with a specific chemotherapy regimen across studies. Solid lines represent direct comparisons. Dashed lines represent indirect comparisons.

The network map is shown in Figure 1. Overall, the studies presented similar inclusion and exclusion criteria – Supplementary Table 1. Patients included in these trials should be 18 years old or above, have the pathological diagnosis of ductal adenocarcinoma and have been submitted to microscopically complete (R0) or incomplete (R1) resection. Also, patients should present an Eastern Cooperative Oncology Group (ECOG) performance status (PS) 0 or 1 at randomisation. However, slight differences among the studies’ protocols were noted. Patients aged 80 years old or above were not included in the PRODIGE 24 study. Also, the ESPAC-4 study allowed for the enrolment of patients with ECOG PS 2. Importantly, both ESPAC-4 and APACT mandated a CT scan at least 3 months before patient registration. Lastly, the PRODIGE 24 and the APACT studies excluded patients with post-operative Carbohydrate antigen 19-9 (CA 19-9) levels above 180 and 100 UI/mL, respectively.

Table 1 describes the characteristics of the studies’ populations. Despite similar inclusion and exclusion criteria, we observed differences in the distribution of patients’ characteristics across trials. We noted significant differences in the rate of microscopically involved resection margins (R1 resection), with a high rate seen particularly in the ESPAC-4 trial. Moreover, patients in the ESPAC-4 trial more often presented with stage III disease. Despite these differences, we considered that, based on the similar inclusion and exclusion criteria among the trials, the populations were homogenous enough for the transitivity assumption to be valid. The assessment of the Brooks–Gelman–Rubin plots suggested model convergence for all comparisons of HRs.

### Disease-free survival

**Figure 2.**
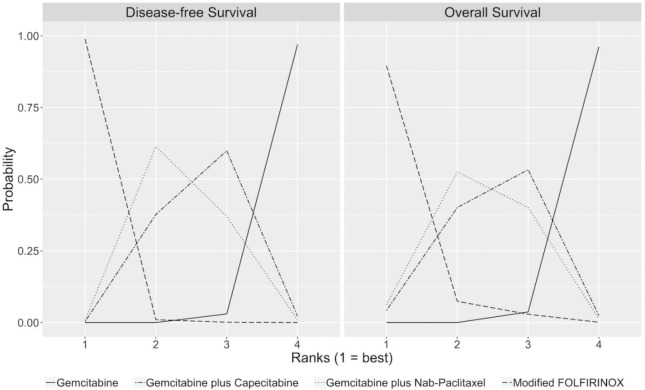
Rankograms for DFS and OS show the probability that each chemotherapy regimen has of being the first, second, third and fourth best chemotherapy regimen in terms of DFS and OS. These rankograms are created by assessing throughout the cycles from the MCMC the relative frequency in which the coefficient of the HR (log HR) for one specific chemotherapy regimen was greater or lesser than those of the other chemotherapy regimens.

The median duration of follow-up in the three studies ranged from 33.6 to 53.3 months. Table 2 describes the survival outcomes of the trials. No significant difference in DFS according to the investigators was observed in ESPAC-4 (*p* = 0.069). Conversely, patients treated with combination chemotherapy experienced improved investigator-assessed DFS in both PRODIGE 24 and APACT. The relative efficacy in terms of DFS of each chemotherapy regimen is shown in Table 3. According to the rankogram, modified FOLFIRINOX had a 98.9% probability of being the most active chemotherapy among the four regimens in the adjuvant setting (Figure 2). The analysis of SUCRA for DFS also described modified FOLFIRINOX as the single most active chemotherapy agent (Table 4). Additionally, GEM-NAB (61.4% probability) was more likely than GEM-CAP (37.7% probability) to be the second best chemotherapy regimen.

### Overall survival

In all three trials, patients treated with combination chemotherapy experienced longer OS. The relative efficacy in terms of OS of each chemotherapy regimen is shown in Table 3. According to the rankogram, modified FOLFIRINOX had 89.6% probability of being the most active chemotherapy among the four regimens in the adjuvant setting (Figure 2). The analysis of SUCRA for OS also described modified FOLFIRINOX as the single most active chemotherapy agent (Table 4). Additionally, GEM-NAB (52.5% probability) was more likely than GEM-CAP (40.1%) to be the second best chemotherapy regimen.

### Outcomes of patients treated with single-agent gemcitabine across trials

There were significant differences in OS across trials for patients undergoing adjuvant treatment with single-agent gemcitabine (Table 2). Patients who were treated in the gemcitabine arm in the ESPAC-4 trial experienced numerically worse OS (median OS: 26.0 months; 95% CI = 22.7–28.4) when compared to those treated with the same regimen in the PRODIGE 24 (median OS: 35.0 months; 95% CI = 28.7–43.9) and APACT (median OS: 37.7 months) trials. However, there were no differences in the relapse-free survival for patients treated with single-agent gemcitabine across the three trials.

### Toxicity

Table 5 describes the toxicity patterns observed in the three studies. In all studies, combination chemotherapy was associated with an increased risk of grade 3–4 toxicity. In ESPAC-4, GEM-CAP was associated with numerically increased rates of grade 3–4 neutropenia, diarrhoea and hand-foot syndrome. In PRODIGE 24, modified FOLFIRINOX was associated with increased rates of grade 3–4 fatigue, nausea, vomiting, diarrhoea and peripheral neuropathy. In the APACT trial, GEM-NAB was associated with higher frequency of grade 3–4 anaemia, fatigue, diarrhoea and peripheral neuropathy. By indirect comparison, patients treated with GEM-NAB experienced the highest rates of grade 3–4 anaemia, neutropenia and peripheral neuropathy. Patients who received modified FOLFIRINOX presented the highest frequency of grade 3–4 diarrhoea. GEM-CAP was the regimen which most commonly led to hand-foot syndrome. However, it is worth noting that when we evaluate only the single-agent gemcitabine arms of the trials, rates of grade 3–4 haematological toxicities were higher in the APACT when compared to the other studies. Thus, indirect comparisons of toxicities across trials should be interpreted with caution.

## Discussion

In our study, modified FOLFIRINOX was the chemotherapy regimen most likely to represent the best currently available option in the adjuvant treatment of pancreatic ductal adenocarcinoma. While the PRODIGE 24 had a lower median follow-up compared to the other two studies and had a rather selected patient population in terms of age and post-operative CA 19-9 levels, modified FOLFIRINOX had 98.9% and 89.6% chance of being the most active chemotherapy regimen in terms of investigator-assessed DFS and OS, respectively. These results are in line with the ones found in a previous Bayesian network meta-analysis including patients with more advanced disease [27]. That said, while long-term survival outcomes of this trial are expected, we believe that FOLFIRINOX is currently the standard of care for all the patients with resected pancreatic cancer who are fit enough to undergo adjuvant chemotherapy with this regimen.

However, some patients will not be optimal candidates to adjuvant treatment with modified FOLFIRINOX. Notably, among the highly selected patient population enrolled in the PRODIGE 24 trial, roughly one-third could not complete the planned 12 cycles of modified FOLFIRINOX; this rate is likely to be even higher among real-world patients. Also, 33% of all patients diagnosed with localised pancreatic cancer in the USA are older than 80 years at diagnosis [29] and it is currently unknown whether this chemotherapy regimen is safe enough for patients in this age group as they were not included in the PRODIGE 24 trial. Moreover, patients with dihydropyrimidine dehydrogenase (DPD) deficiency (present in 8% of African-American and 3–5% of Caucasian patients) [30] are at increased risk of toxicity when treated with fluoropyrimidines while those with uridine diphosphate glucoronosyltransferase 1A (UGT1A) polymorphisms (present in at least 20% of Caucasians and 15% of African-Americans or Asian patients) [31] are at an increased risk of toxicity when treated with irinotecan. While adjustments in the dose of irinotecan seem to be sufficient to decrease the risk of severe toxicity even in patients with homozygous UGT1A polymorphism [32], treatment with a fluoropyrimidine in patients with severe DPD deficiency is associated with a prohibitively high risk of severe toxicity and is not recommended [33]. Additionally, other limitations associated with the use of FOLFIRINOX include the need for a permanent catheter and the cumulative cold-induced peripheral neuropathy (particularly relevant in regions with long winters).

Apart from toxicity concerns, recent data points out that the efficacy of 5-Fluorouracil-based chemotherapy in pancreatic cancer might depend on the patterns of tumour gene expression. GATA6 is a transcription factor associated with cell differentiation in early pancreatic embryogenesis and low levels of GATA6 are associated with basal-like (or quasi-mesenchymal) gene expression profile. While no prospective randomised trial in the adjuvant setting has been specifically designed to assess this hypothesis, a retrospective analysis from the ESPAC-3 trial showed that patients who had tumours with low or medium levels of GATA6 expression who were treated with 5-Fluorouracil had worse survival when compared to those with high-level tumours [34]. The same phenomenon was not seen for those treated with gemcitabine. Also, pre-clinical data have shown that gemcitabine treatment might be more active against tumours with basal-like (or quasi-mesenchymal) gene expression profile (and therefore, low levels of GATA6 expression) [35] and recent data in the metastatic setting support the effects of this treatment interaction [36]. Thus, while GATA6 expression and gene expression profiling are not currently used to tailor adjuvant chemotherapy for pancreatic cancer, compelling emerging data suggest the choice of fluoropyrimidine-based or gemcitabine-based chemotherapy could be guided by the molecular characteristics of the tumour.

The use of gemcitabine-based doublets could match the need for less toxic or non-fluoropyrimidine-based regimens in the adjuvant setting. In this sense, we showed that GEM-NAB is likely associated with the highest probability of being the best gemcitabine-based treatment in terms of both investigator-assessed DFS and OS. GEM-CAP had a slightly worse performance than GEM-NAB in terms of survival outcomes. Also, in the real world, the use of GEM-CAP has been shown to be challenging given its toxicity profile [37]. Likewise, the rate of chemotherapy completion among patients treated with GEM-CAP in the ESPAC-4 study was the lowest among the three studies – Supplementary Table 2. Furthermore, retrospective data from the X-ACT study in colon cancer suggest worse tolerance to fluoropyrimidine among North Americans when compared to Europeans [38]. Because only European patients were included in the ESPAC-4, non-European Western patients might face even more difficulties in trying to complete adjuvant chemotherapy with GEM-CAP. However, despite potential differences in the chances of experiencing chemotherapy-related toxicity between North Americans and Europeans, it is important to state that current data support similar survival rates for patients with stage I–II pancreatic cancer who undergo resection in the United States and in Europe [39], which suggest that potential differences in survival outcomes across these trials are probably not secondary to ethnic or geographical reasons.

Despite these results, one might say that GEM-NAB should not be considered a treatment option in this setting since the APACT trials failed to meet the primary endpoint of DFS as assessed by the independent review. We believe that the conclusion as to whether a trial is considered positive or negative is complex and should not rely solely on the statistical significance of the test for the primary outcome [40]. The APACT investigators chose the independently reviewed DFS as the primary outcome in an attempt to improve reproducibility. Nonetheless, pancreatic cancer relapse is often suspected based on clinical (symptoms) or laboratory (CA 19-9 levels) information, which is not available for the independent reviewers [41, 42]. These data seem particularly important for patients with locoregional recurrences, which might be very difficult to distinguish from post-operative alterations based only on radiological grounds [43, 44]. Accordingly, a recent analysis from the APACT trial showed that there were significant discordances in the number of DFS events between independent and investigator assessment that likely influenced outcomes [21].

Another argument that supports the use of GEM-NAB in the adjuvant setting is the significant OS benefit. This outcome is perceived by many experts in pancreatic cancer as the most important indicator of outcome quality [45]. Accordingly, OS was the primary outcome for both the PRODIGE 24 and the ESPAC-4 trials. Interestingly, in the latter study, the OS benefits were considered significant despite the lack of significant improvement in DFS. Additionally, in the recently presented SWOG S1505 trial [46], which compared perioperative modified FOLFIFIRINOX to perioperative GEM-NAB in the resectable disease setting, no significant differences in DFS and OS times were found. That said, despite the limited sample size and some methodological issues, the results of SWOG S1505 suggest that FOLFIRINOX and GEM-NAB offer similar anti-tumour activity in localised pancreatic cancer, further supporting the use of adjuvant GEM-NAB in case modified FOLFIRINOX is not feasible.

Patients treated with single-agent gemcitabine in the ESPAC-4 trial experienced numerically worse OS when compared to those in the PRODIGE24 and APACT trials. That is in line with the distribution of known prognostic factors, such as ECOG and stage, across these studies. This might have favoured the modified FOLFIRINOX and GEM-NAB arms when compared to GEM-CAP, at least in terms of absolute survival benefit. Furthermore, the lack of seemly significant differences in median relapse-free survival across the single-agent gemcitabine arms in these trials suggests that factors apart from the adjuvant chemotherapy regimen might have played a part in determining OS, such as characteristics of the post-progression therapy.

Our study has limitations. We did not carry out a systematic search for all the articles comparing different adjuvant chemotherapy regimens for patients with resected pancreatic cancer. Nevertheless, apart from S-1 which is used almost exclusively in Asia, it is largely known by those with expertise in pancreatic cancer that these are the most clinically relevant chemotherapy regimens tested in randomised trials in the adjuvant setting. Furthermore, inconsistencies in the descriptions of the toxicities prevented us from making indirect comparisons of grade 3–4 or severe adverse events. However, we displayed the data from the studies in a way that readers can have an idea of the comparative risks of toxicities. Our study also has merits. As far as we know, this is the first Bayesian network meta-analysis evaluating the comparative effectiveness of these chemotherapy regimens. With this methodology, we were able to rank the efficacy of these regimens using a probabilistic approach that can aid the decision-making process. Moreover, we used matched outcomes, considering differences in independent reviewer-assessed and investigator-assessed DFSs. Furthermore, we present updated results for survival analysis from two of the three studies.

## Conclusion

Modified FOLFIRINOX is the standard of care for the treatment of pancreatic cancer in the adjuvant setting for patients fit enough to receive this chemotherapy regimen. For those with contraindications or who are not fit enough for modified FOLFIRINOX, GEM-NAB might be the preferred adjuvant therapy, particularly to those patients who have contraindications for fluoropyrimidines.

## Conflicts of interest

Victor Hugo Fonseca de Jesus received honoraria from United Medical and had travel expenses paid by United Medical in the past 12 months. United Medical is the company responsible for the distribution and sale of nab-paclitaxel in Brazil. Rachel Pimenta Riechelmann received consultancy fees from Astra Zeneca in the past 12 months.

## Funding source

This study had no funding source.

## Authors’ contributions

Victor Hugo Fonseca de Jesus: conceptualisation, methodology, data curation, data analyses, writing and visualisation; Rachel Pimenta Riechelmann: methodology, writing and visualisation.

## Figures and Tables

**Supplementary Table 1.** Inclusion and exclusion criteria.

	ESPAC-4	PRODIGE 24	APACT
**Inclusion**			
Age (years)	≥18	18–79	≥18
Histology	Ductal adenocarcinoma	Ductal adenocarcinoma	Ductal adenocarcinoma
Extent of resection	R0^a^ or R1	R0^a^ or R1	R0 or R1
Time from surgery (weeks)	-	3–12	<12
Staging	Non-metastatic	Non-metastatic^b^	I or II^c^
Performance status (ECOG)	0–2	0 and 1	0 and 1
Organ function	Adequate haematologic, renal and hepatic functions	Adequate haematologic, renal and hepatic functions	-
**Exclusion**			
Post-operative tumor marker (U/mL)	No restriction	>180	>100
Previous treatment	Radiotherapy or chemotherapy	Radiotherapy or chemotherapy	Radiotherapy or chemotherapy
Comorbidity	-	Symptomatic coronary artery disease or congestive heart failure	-

a Defined as no cancer cells within 1 mm of all resection margins

b Clear CT scan of chest, abdomen, and pelvis required within 3 months before randomization

c CT without evidence of disease

**Supplementary Table 2.** Treatment compliance.

	ESPAC-4		PRODIGE 24		APACT	
	Gemcitabine	Gemcitabine plus Capecitabine	Gemcitabine	Modified FOLFIRINOX	Gemcitabine	Gemcitabine plus Nab-Paclitaxel
	*N* = 366	*N* = 364	*N* = 246	*N* = 247	*N* = 423	*N* = 429
**Dose intensity – %** Gemcitabine Capecitabine 5-Fluorouracil Oxaliplatin Irinotecan Nab-Paclitaxel	93.0 - - - - -	83.0 78.0 - - - -	- - - - - -	- - - - - -	91.2 - - - - -	80.0 - - - - 75.1
**Treatment completion (%)** **Yes** **No**	239 (65.3) 127 (34.7)	195 (53.6) 169 (46.4)	192 (79.0) 54 (21.0)	158 (66.4) 89 (33.6)	300 (70.9) 123 (29.1)	283 (66.0)^a^ 146 (34.0)

a In cycle 6, 59% of patients on Gemcitabine plus Nab-Paclitaxel received Nab-Paclitaxel

**Table 1.** Characteristics of the studies’ populations.

	ESPAC-4		PRODIGE 24		APACT		p value
	Gemcitabine	GEM-CAP	Gemcitabine	Modified FOLFIRINOX	Gemcitabine	GEM-NAB	
	*N* = 366 (%)	*N* = 364 (%)	*N* = 246 (%)	*N* = 247 (%)	*N* = 434 (%)	*N* = 432 (%)	
**Sex** Male Female	212 (57.9) 154 (42.1)	202 (55.5) 162 (44.5)	135 (54.9) 111 (45.1)	142 (57.5) 105 (42.5)	253 (58.3) 181 (41.7)	228 (52.8) 204 (47.2)	0.894^b^
**Age (years)** Median Range	65 37–80	65 39–81	64 (30–81)	63 (30–79)	64 38–86	64 34–83	-
**ECOG PS^a^** 0 1 2 Unknown	158 (43.2) 199 (54.4) 9 (2.4) 0 (0.0)	150 (41.2) 202 (55.5) 12 (3.3) 0 (0.0)	127 (51.6) 115 (46.7) 0 (0) 4 (1.6)	122 (49.4) 123 (49.8) 0 (0) 2 (<1)	268 (61.8) 166 (38.2) 0 (0.0) 0 (0.0)	252 (58.3) 180 (41.7) 0 (0.0) 0 (0.0)	<0.001^b^
**Preoperative CA 19-9 (UI/mL)** Median Range	142.5 0.9–10761	154.5 0.8–76549	- -	- -	- -	- -	-
**Postoperative CA 19-9 (UI/mL)** Median Range	20.5 0.1–2448.3	17.6 0.6–8112	- -	- -	12.9 -	14.31 -	-
**Resection margin** R0 resection R1 resection	147 (40.2) 219 (59.8)	143 (39.3) 221 (60.7)	134 (54.5) 112 (45.5)	148 (59.9) 99 (40.1)	334 (77.0) 100 (23.0)	327 (75.7) 105 (24.3)	<0.001^b^
Tumour grade Well differentiated Moderately differentiated Poorly differentiated or undifferentiated Unknown	30 (8.2) 192 (52.5) 142 (38.8) 2 (0.5)	32 (8.8) 175 (48.1) 149 (40.9) 8 (2.2)	79 (32.1) 125 (50.8) 29 (11.8) 13 (5.3)	70 (28.3) 124 (50.2) 35 (14.2) 18 (7.3)	55 (12.7) 241 (55.5) 117 (27.0) 21 (4.8)	49 (11.3) 264 (61.1) 102 (23.6) 17 (3.9)	<0.001^c^
**Lymph nodes** Negative Positive	67 (18.3) 299 (81.7)	76 (20.9) 288 (79.1)	68 (27.6) 178 (72.4)	59 (23.9) 188 (76.1)	122 (28.1) 312 (71.9)	121 (28.0) 311 (72.0)	<0.001^b^
**Maximum tumour size (mm)** Median Range	30 0–110	30 6–105	30 6–120	30 8–90	- -	- -	
**Tumour stage (AJCC 7th Edition)** I II III IV	7 (1.9) 29 (7.9) 325 (88.8) 5 (1.3)	15 (4.1) 20 (5.5) 326 (89.6) 3 (0.8)	14 (5.7) 226 (91.9) 1 (0.4) 5 (2.0)	12 (4.9) 226 (91.5) 1 (0.4) 8 (3.2)	- - - -	- - - -	<0.001^b^
**Surgery** Whipple resection Total pancreatectomy Pylorus-preserving resection Distal pancreatectomy	188 (51.4) 27 (7.4) 122 (33.3) 29 (7.9)	182 (50.0) 22 (6.0) 129 (35.4) 31 (8.5)	190 (77.2) - - -	202 (81.8) - - -	- - - -	- - - -	-
**Venous resection** No Yes Unknown	298 (81.4) 63 (17.2) 5 (1.4)	323 (88.7) 39 (10.7) 2 (0.5)	176 (71.5) 69 (28.0) 1 (<1)	192 (77.7) 53 (21.5) 2 (<1)	- - -	- - -	<0.001^b^
**Lymphovascular invasion** No Yes Unknown	- - -	- - -	79 (32.1) 135 (54.9) 32 (13.0)	55 (22.3) 154 (62.3) 38 (15.4)	- - -	- - -	-
**Perineural invasion** No Yes Unknown	- - -	- - -	24 (9.8) 207 (84.1) 15 (6.1)	16 (6.5) 205 (83.0) 26 (10.5)	- - -	- - -	-

a ECOG PS: Eastern Cooperative Oncology Group (ECOG) performance status

b Based on Fisher’s exact test; only for categorical variables

c Based on chi-square test; only for categorical variables

**Table 2.** Survival outcomes according to the treatments across studies.

	ESPAC-4		PRODIGE 24		APACT	
	Gemcitabine	GEM-CAP	Gemcitabine	Modified FOLFIRINOX	Gemcitabine	GEM-NAB
	*N* = 366	*N* = 364	*N* = 246	*N* = 247	*N* = 434	*N* = 432
Median follow-up – months (95% CI)	43.2^a^ (39.7–45.5)		33.6 (30.3–36.0)		53.0 -	53.3 -
**DFS**						
Median DFS – months (95% CI)	13.4 (11.7–15.5)	14.2 (12.2–16.9)	12.8 (11.7–15.2)	21.6 (17.7–27.6)	13.7	16.6
HR (95% CI)	1.0	0.85 (0.73–1.00)	1.0	0.58 (0.46–0.73)	1.0	0.82 (0.69–0.97)
**OS**						
Median OS – months (95% CI)	26.0 (22.7–28.4)	27.7 (23.3–31.2)	35.0 (28.7–43.9)	54.4 (41.8–NA)	37.7 -	41.8 -
HR (95% CI)	1.0	0.84 (0.70–0.99)	1.0	0.64 (0.48–0.86)	1.0	0.82 (0.69–0.97)

a Based on original publication (not on updated analysis). For the ESPAC-4, administrative censoring occurred at 5 years of follow-up

CI, Confidence interval; NA, not reached

**Table 3.** HRs and 95% CrIs for DFS and OS.

DFS				
	Gemcitabine	GEM-CAP	GEM-NAB	Modified FOLFIRINOX
Gemcitabine				
GEM-CAP	0.85 (0.73–0.99)			
GEM-NAB	0.82 (0.70–0.97)	0.97 (0.77–1.21)		
Modified FOLFIRINOX	0.58 (0.46–0.73)	0.69 (0.52–0.90)	0.72 (0.53–0.94)	
**OS**				
	Gemcitabine	GEM-CAP	GEM-NAB	Modified FOLFIRINOX
Gemcitabine				
GEM-CAP	0.84 (0.71–1.00)			
GEM-NAB	0.82 (0.69–0.97)	0.98 (0.76–1.25)		
Modified FOLFIRINOX	0.65 (0.48–0.86)	0.77 (0.54–1.07)	0.79 (0.56–1.10)	

**Table 4.** SUCRA for DFS and OS.

Chemotherapy	DFS	OS
Modified FOLFIRINOX	0.996	0.955
GEM-NAB	0.540	0.546
GEM-CAP	0.454	0.487
Gemcitabine	0.010	0.013

**Table 5.** Toxicity patterns of studies’ treatments.

	ESPAC-4		PRODIGE 24		APACT	
	Gemcitabine	GEM-CAP	Gemcitabine	Modified FOLFIRINOX	Gemcitabine	GEM-NAB
	*N* = 366 (%)	*N* = 359 (%)	*N* = 243 (%)	*N* = 238 (%)	*N* = 423 (%)	*N* = 429 (%)
**All Grade 3–4**	196 (53.6)	226 (63.0)	128 (52.9)	180 (75.9)	286 (67.6)	371 (86.5)
**Haematological**						
**Anaemia** All grades Grades 3–4	- 14 (3.8)	- 8 (2.2)	216 (89.3) 6 (2.5)	200 (84.7) 8 (3.4)	- 33 (7.8)	- 63 (14.7)
**Neutropenia** All grades Grades 3–4	- 89 (24.3)	- 137 (38.2)	154 (63.6) 63 (26.0)	157 (66.5) 67 (28.4)	- 184 (43.5)	- 212 (49.4)
**Febrile neutropenia** Grade 3–4	-	-	10 (4.1)	7 (3.0)	21 (5.0)	4 (0.9)
**Thrombocytopenia** All grades Grades 3–4	- 7 (1.9)	- 8 (2.2)	122 (50.4) 11 (4.5)	111 (47.0) 3 (1.3)	- -	- -
**Lymphopenia** All grades Grades 3–4	- 11 (3.0)	- 9 (2.5)	117 (48.3) 7 (2.9)	87 (36.9) 3 (1.3)	- -	- -
**Non-haematological**						
**Fatigue** All grades Grades 3–4	- 19 (5.2)	- 20 (5.6)	187 (77.6) 11 (4.6)	199 (84.0) 26 (11.0)	- 13 (3.1)	- 43 (10)
**Nausea** All grades Grades 3–4	- -	- -	133 (5.2) 2 (0.8)	187 (78.9) 13 (5.5)	- -	- -
**Vomiting** All grades Grades 3–4	- -	- -	70 (29.0) 3 (1.2)	108 (45.6) 12 (5.1)	- -	- -
**Diarrhoea** All grades Grades 3–4	- 6 (1.6)	- 19 (5.3)	118 (49.0) 9 (3.7)	200 (84.4) 44 (18.6)	- 4 (0.9)	- 22 (5.1)
**Mucositis** All grades Grades 3–4	- -	- -	36 (14.9) 0 (0.0)	80 (33.8) 6 (2.5)	- -	- -
**Hand-foot syndrome** All grades Grades 3–4	- 0 (0.0)	- 26 (7.2)	2 (0.8) 0 (0)	12 (5.1) 1 (0.4)	- -	- -
**Peripheral neuropathy** All grades Grades 3–4	- -	- -	21 (8.7) 0 (0.0)	145 (61.2) 22 (9.3)	- 0 (0.0)	- 64 (14.9)
**Alopecia** All grades	-	-	47 (19.5)	64 (27.0)	-	-
**Thrombosis** All grades Grades 3–4	- 9 (2.5)	- 8 (2.2)	19 (79) 1 (0.4)	14 (5.9) 6 (2.5)	- -	- -

## Supplementary material

### Codes for the WinBUGS

**# MODEL for OS**

***# Defining the model for log-hazard ratios (lhr)***

model {

for(i in 1:ndp){

prec[i]<- 1/(se[i]*se[i])

lhr[i]~dnorm(md[i],prec[i])

***# Defining the fixed-effect model for the log hazard ratios (lhr)***

md[i] <- d[t[i]] - d[b[i]]

**# Give priors to *log hazard ratios (lhr)***

d[1]<-0

for (k in 2:nt){

d[k] ~ dnorm(0,.001)

}

***# Defining ranks (rk)***

for(k in 1:nt){

rk[k]<- rank(d[],k)

best[k]<-equals(rk[k],1)

**# Defining SUCRA**

for(j in 1:nt){effectiveness[k,j]<-equals(rk[k],j)}}

for(k in 1:nt){for(j in 1:nt){cumeffectiveness[k,j]<-sum(effectiveness[k,1:j])}}

for(k in 1:nt){SUCRA[k]<-sum(cumeffectiveness[k,1:(nt-1)])/(nt-1)

}

**# Construct all pairwise log hazard ratios (lhr) and hazard ratios (HR)**

for (c in 1:nt-1){

for (k in (c+1):nt){

lhzr[c,k] <- d[k] - d[c]

HR[c,k] <- exp(lhzr[c,k])

}

}

}

***# Entering the data in the model (observed data)***

data #########

list(ndp=3, nt=4, t=c(2,3,4), b=c(1,1,1), lhr=c(-0.174353387,-0.198450939,-0.446287103), se=c(0.088424645,0.088788212,0.148761807))

#########

**# Entering initial values in the model**

initials # three chains list(d=c(NA,-0.5,-0.5,-0.5))

list(d=c(NA,0,0,0)

list(d=c(NA,0.5,0.5,0.5))

**# MODEL FOR DFS**

***# Defining the model for log-hazard (lhr)***

model {

for(i in 1:ndp){

prec[i]<- 1/(se[i]*se[i])

lhr[i]~dnorm(md[i],prec[i])

***# Defining the fixed-effect model for the log hazard ratios (lhr)***

md[i] <- d[t[i]] - d[b[i]]

**# Give priors to *log hazard ratios (lhr)***

d[1]<-0

for (k in 2:nt){

d[k] ~ dnorm(0,.001)

}

***# Defining ranks (rk)***

for(k in 1:nt){

rk[k]<- rank(d[],k)

best[k]<-equals(rk[k],1)

**# Defining SUCRA**

for(j in 1:nt){effectiveness[k,j]<-equals(rk[k],j)}}

for(k in 1:nt){for(j in 1:nt){cumeffectiveness[k,j]<-sum(effectiveness[k,1:j])}}

for(k in 1:nt){SUCRA[k]<-sum(cumeffectiveness[k,1:(nt-1)])/(nt-1)

}

**# Construct all pairwise log hazard ratios (lhr) and hazard ratios (HR)**

for (c in 1:nt-1){

for (k in (c+1):nt){

lhzr[c,k] <- d[k] - d[c]

HR[c,k] <- exp(lhzr[c,k])

}

}

}

***# Entering the data in the model (observed data)***

data #########

list(ndp=3, nt=4, t=c(2,3,4), b=c(1,1,1), lhr=c(-0.1625189,-0.1984509,-0.5447272), se=c(0.08028335,0.08409595,0.11781073))

#########

**# Entering initial values in the model**

initials # three chains list(d=c(NA,-0.5,-0.5,-0.5))

list(d=c(NA,0,0,0)

list(d=c(NA,0.5,0.5,0.5))

### Formulas

The formula below specifies the Bayesian model:

**lhr_i_ ~ N (θ_i_,V_i_)**

where **lhr_i_** represents trial-specific log hazard ratio and **V_i_** represents the within-trial variance. It is worth noting that in WinBUGS variance needs to be recorded as precision (precision being the inverse of the variance). As stated in the Methods section, we employed a pragmatic approach in which we fit a fixed-effect model to the data. In this setting, any difference between the log hazard ratios is believed to be due to sampling error [1]. In other words:

**lhr_i_ = θ + ε_i_**

where the sampling error **ε_i_** is normally distributed with mean 0 and variance equal to the within-trial variance. This fixed-effect model can be described as:

**θ_i_ = d_ti_ - d_bi_**

where **d_bi_** are defined as basic parameters and **d_ti_** are defined as functional parameters. For the basic parameters, a non-informative prior with mean 0 and variance 1000 was chosen. Further information can be found on the paper by Hu *et al* [2]
